# Protein Sequence Annotation Tool (PSAT): a centralized web-based meta-server for high-throughput sequence annotations

**DOI:** 10.1186/s12859-016-0887-y

**Published:** 2016-01-20

**Authors:** Elo Leung, Amy Huang, Eithon Cadag, Aldrin Montana, Jan Lorenz Soliman, Carol L. Ecale Zhou

**Affiliations:** Computing Applications and Research, Global Security Computing Applications Division, Lawrence Livermore National Security, Livermore, CA 94550 USA; Personalis, Menlo Park, CA 94025 USA; Capella Biosciences, Palo Alto, CA USA; LinkedIn, Mountain View, CA 94043 USA

## Abstract

**Background:**

Here we introduce the Protein Sequence Annotation Tool (PSAT), a web-based, sequence annotation meta-server for performing integrated, high-throughput, genome-wide sequence analyses. Our goals in building PSAT were to (1) create an extensible platform for integration of multiple sequence-based bioinformatics tools, (2) enable functional annotations and enzyme predictions over large input protein fasta data sets, and (3) provide a web interface for convenient execution of the tools.

**Results:**

In this paper, we demonstrate the utility of PSAT by annotating the predicted peptide gene products of *Herbaspirillum sp.* strain RV1423, importing the results of PSAT into EC2KEGG, and using the resulting functional comparisons to identify a putative catabolic pathway, thereby distinguishing RV1423 from a well annotated *Herbaspirillum* species. This analysis demonstrates that high-throughput enzyme predictions, provided by PSAT processing, can be used to identify metabolic potential in an otherwise poorly annotated genome.

**Conclusions:**

PSAT is a meta server that combines the results from several sequence-based annotation and function prediction codes, and is available at http://psat.llnl.gov/psat/. PSAT stands apart from other sequence-based genome annotation systems in providing a high-throughput platform for rapid de novo enzyme predictions and sequence annotations over large input protein sequence data sets in FASTA. PSAT is most appropriately applied in annotation of large protein FASTA sets that may or may not be associated with a single genome.

**Electronic supplementary material:**

The online version of this article (doi:10.1186/s12859-016-0887-y) contains supplementary material, which is available to authorized users.

## Background

Advances in next generation sequencing technologies have enabled rapid generation of newly sequenced genomes at a rate that can no longer be handled by a single-core non-distributed computing system in a feasible manner [1, 2]. The large volume of sequencing data that are now available has created profound challenges in data transfer and analysis [3]. High throughput computing on supercomputers was recently introduced to meet these challenges [4, 5]. However, high performance computing can be costly, and access to a supercomputing facility can be limited for small laboratories.

In recent years, a number of publicly available meta-servers have been developed for protein sequence annotation [6–8], but public access to these servers is often restricted to a limit that ranges from 1 to 10 protein sequences per HTTP request. Other similar servers that allow users to submit their whole genome include the IGS microbial annotation pipeline [9] and the Integrated Microbial Genomes [10]. While these servers provide the convenience of a whole genome annotation, they do not accept protein sequences as input and, therefore, cannot run analyses on a set of pre-selected proteins from a given genome or a set of un-related proteins from multiple genomes. On the other hand, a local server [11] that is custom-built by using the available source code provides the option to customize the input size and to avoid all possible public exposure of private data. However, building such a local server requires expert knowledge and can be time consuming.

In this paper, we describe a new high-throughput, genome-wide analysis tool for deriving enzymatic functions and other annotations for protein sequences. Many tools and databases have already been developed to address the need of enzyme function annotations [12]. However a publicly available, high-throughput meta-server is needed to combine the existing annotation tools from their disparate domains in efforts to support genome-scale sequence annotations, whereby a single-user interface can be used to access a variety of computational tools and the results from these tools. Here, we present the Protein Sequence Annotation tool (PSAT), a web-based, sequence annotation meta-server for performing integrated, high-throughput, genome-wide sequence analyses. Our goals in building PSAT were to (1) create an extensible platform for integration of multiple sequence-based bioinformatics tools, (2) enable functional annotations and enzyme predictions over large input protein fasta data sets, and (3) provide a web interface for convenient execution of the tools. In this paper, we demonstrate the versatility of PSAT by inferring the potential metabolic pathways of a draft genome – *Herbaspirillum sp*. strain RV1423.

## Implementation

### Server-side architecture

**Fig. 1:**
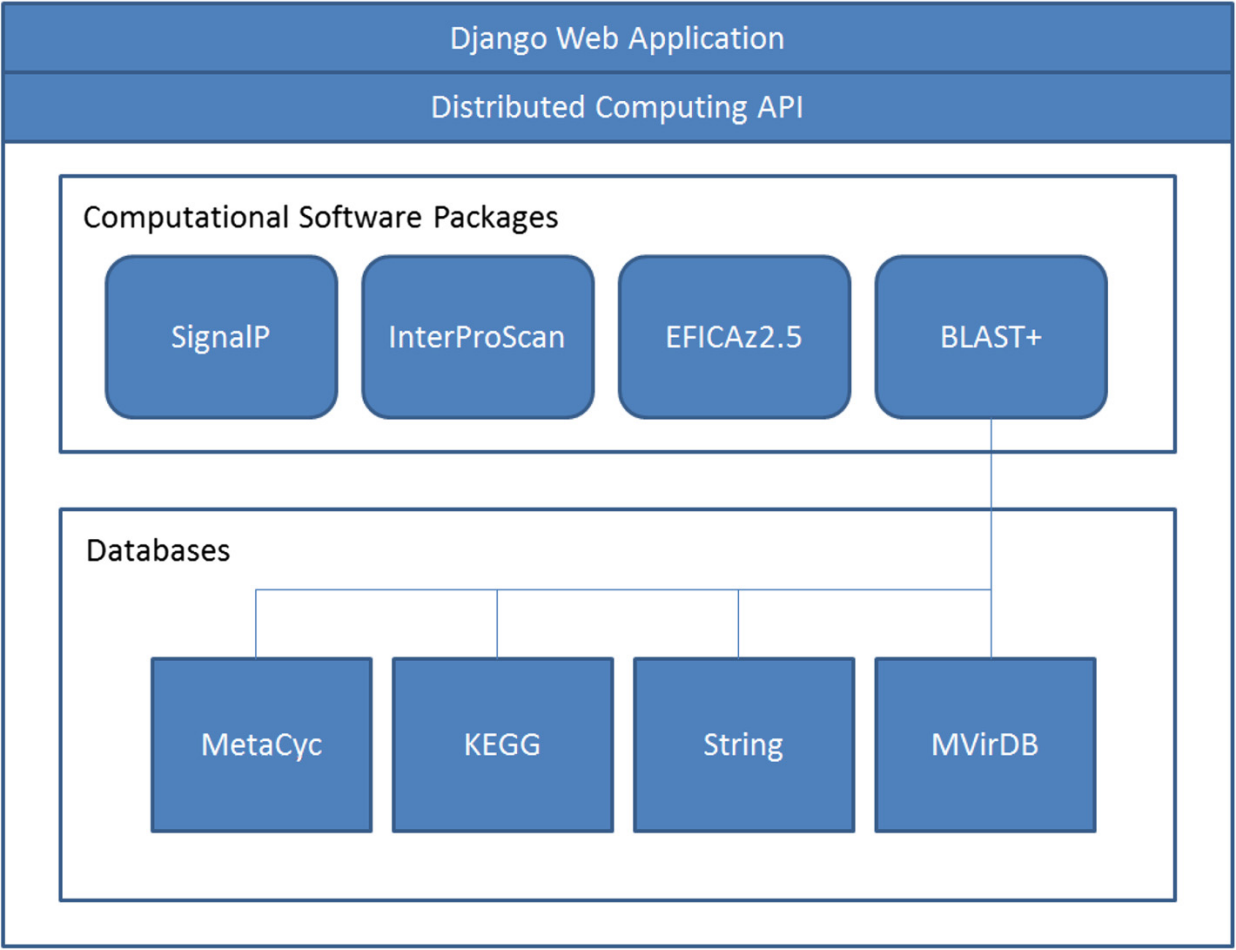
PSAT web-based architecture: currently available computational sources for sequence annotations

An overview of the PSAT architecture is shown in Fig. 1. As of this writing, the PSAT back end is implemented in Python 2.6 on a Redhat Linux cluster comprising 8 Dual Intel Xeon X5690 6-core, 3.46GHz processors and 1 TB disk drive. Data pertaining to job management are managed using Python SQLite, and persistent storage is managed using MySQL. Once the web server accepts a PSAT job requested by a user, it communicates with the distributed computing layer to perform all computing tasks relevant to the PSAT job. These computing tasks executed through the distributed computing APIs may involve any or all of the software packages currently supported by PSAT, which are InterProScan [13], SignalP [14], EFICAz [15], and BLAST+ [16]. In our architecture, a software package may invoke any of the databases installed on the PSAT server, including KEGG [17], MetaCyc [18], STRING [19] and MVirDB [20]. Currently, only Blast + can invoke these databases as it is the only annotation tool installed on PSAT designed to search through a local but nonspecific biological sequence database. The KEGG data set was acquired from the Kanehisa laboratory through an organizational license. Current versions of these codes and data sources can be found on the PSAT home page (http://psat.llnl.gov/psat/).

### Django web application

PSAT is powered by Django, which is a web application framework available through open source [21]. The Django web framework adopts the standard “model, view, controller (MVC)” architectural pattern, whereby the model defines the data, the view controls data presentation to the user, and the controller depends on the model and the view to perform the necessary operations on data when interpreting a user request [22]. Using such a framework in our web application removes the dependencies between the model and view, which in turn enhances source code reusability and software stability [23]. In our Django web application, we can add a new database or a new software package support without modifying a significant portion of our source code.

### Distributed computing

**Fig. 2:**
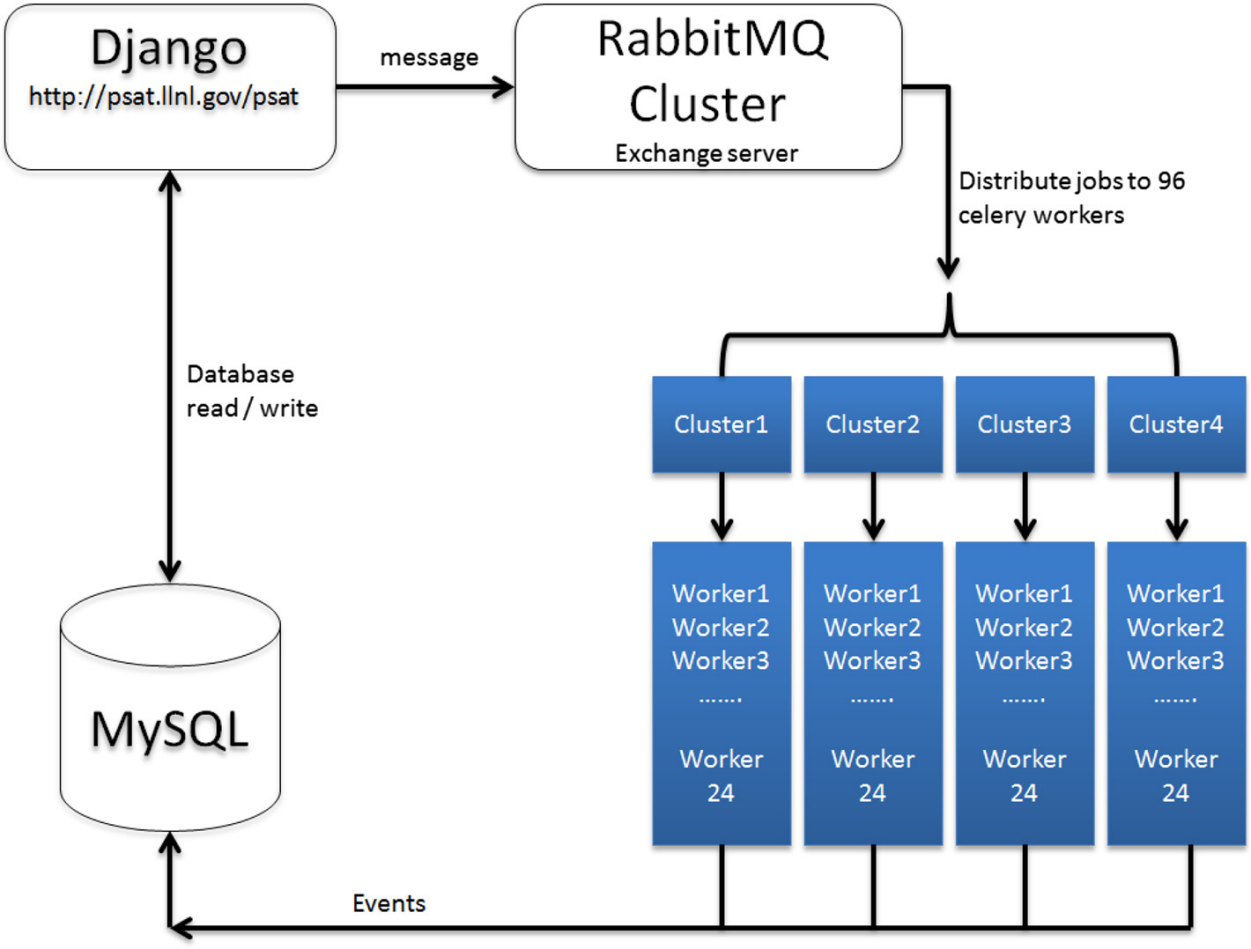
Real-time messaging system between the Django web application, RabbitMQ, Celery workers, and MySQL database

All tasks related to parallel computing in PSAT are handled by Celery [24] and RabbitMQ [25]. RabbitMQ is an open-source message broker software, which offers robust, highly scalable asynchronous processing [25], whereas Celery distributes the jobs to its workers as it consumes the message sent by RabbitMQ [24]. Fig. 2 shows the real-time messaging system between the Django web application, RabbitMQ, Celery workers, and the local MySQL database. When the backend Django web application receives a job request, Celery creates a task queue to wrap up the job execution function through its decorator, and pushes that to the RabbitMQ server. RabbitMQ acts as an exchange server distributing the jobs to 64 celeryd workers over 8 compute nodes, based on processor availability. When a celeryd worker receives a job request, the worker processes it right on the node. All the job processing is handled asynchronously in the background without causing front-end web page delay (hanging). On the server side, data provenance is captured to ensure that sequence analysis results are generated in a reproducible and systematic manner.

Each core of the Linux cluster can serve as a celery worker to perform a specific computational task. Jobs executed on the 64 cores are run in parallel and are distributed into multiple subtasks handled by multiple computing threads. When sequence analysis by all celery worker threads is complete in the background, PSAT automatically combines the job results for user download through the PSAT website or a link specified in a notification email.

### PSAT package support

PSAT provides a centralized computational resource for a variety of protein sequence annotation tools. PSAT supports a suite of software packages designed to predict enzyme functions for a given set of protein sequences, most notably EFICAz 2.5, which uses machine learning algorithms to automatically infer the enzyme function of a protein [15]. MetaCyc Blast and KEGG Blast are also available to derive similar information by running BLAST+ against the open-source MetaCyc and licensed KEGG databases [17, 18], respectively. Combining the results of EFICAz, MetaCyc blast, and KEGG blast analyses produces lists of Enzyme Commission (EC) numbers that putatively describe the functions of the query proteins. In a summary output file, for each protein, all predicted EC numbers are listed numerically followed by the evidence (i.e., EFICAz, KEGG blast, MetaCyc blast) for that EC. No attempt is made to rank order the evidence items or to combine them into a single prediction. The PSAT output enables comparison of annotation results across different annotation methods. The predicted EC numbers are then linked to metabolic pathways by means of a RESTful interface to the KEGG API [26] to retrieve up-to-date enzyme-to-pathway mappings.

To supplement the primary goal of whole proteome enzyme function prediction of PSAT, we have also included functional annotation codes, SignalP 4.0 [14] and InterProScan 5 [13], to the meta-server. Furthermore, the String [19] and MVirDB [20] databases are now also available for a BLAST+ search on PSAT.

### User interfaces and access

PSAT was built using a thin-client approach, in which the entire MVC logic resides on the server side. Hence, only a web browser is required in order to run a sequence analysis on PSAT. An online registration for a user account is available for all PSAT users at http://psat.llnl.gov/psat. User authentication is required in order to submit annotation jobs to the PSAT server. When submitting a new job, a user is required to either copy the fasta sequences onto the job submission form or upload a file containing a set of amino acid sequences in fasta format. Then, when the job is finished, an automated email with a job result download link is sent to the user.

Summary results are presented as a tabbed text file containing computational predictions and reliability metrics from the set of tools that were run for a given job. Because the user’s input FASTA sequences are processed by PSAT in parallel, individual computations will finish out of order with respect to the original input FASTA file. Therefore, prior to processing, the user’s headers are pre-pended with a sequential numeric identifier to enable re-establishment of the original ordering upon completion of the job. The (voluminous) raw data results are stored on our server in persistent storage and are available upon request for up to 3 months following completion of a job.

All PSAT users must login using their credentials at the beginning of each PSAT session. Once a PSAT user has successfully logged in, a homepage will be displayed dynamically, showing a list of the recently submitted PSAT jobs by the authenticated user and the corresponding job status. In order to control server load and file transfer volumes, we limit the number of submitted protein sequences to 800. However, users are encouraged to contact the authors regarding submission of jobs in excess of 800, and we welcome jobs that require enzyme prediction over whole bacterial proteomes or non-specific protein sets involving up to 10,000 protein sequences per job.

### Genomic sequence for the case study

The genome of *Herbaspirillum* sp. strain RV1423 (henceforth RV1423), which was isolated from underground water contaminated with alkane and aromatic hydrocarbons, has already been sequenced in a whole-genome shotgun project [27]. The draft genome of RV1423 obtained from NCBI [28] comprises 131 contigs under the accession numbers CBXX010000001 to CBXX010000131. This newly sequenced genome, which has been reported previously to potentially degrade naphthalene [27], was selected for our case study to demonstrate the ability of PSAT to derive functional annotations and link them to metabolic pathways that may be present in a draft genome that has not yet been fully annotated.

### Pre-processing of genomic sequence

A previous study has identified a set of 5732 potential protein-coding genes in RV1423 by using the RAST server version 4.0 [27]. A renumbered and newer version of RAST server 2.0 [29, 30] was used in our study, and generated a set of 5649 features that are potentially protein-coding genes. These predicted genomic features were translated to amino acid sequences, which served as input for PSAT. EC data arising from the PSAT processing were subsequently re-formatted for input to EC2KEGG [31].

### EC2KEGG analysis and statistical significance

The pathways inferred from results generated by PSAT may be over- or under-represented when compared to a reference genome. To evaluate the statistical significance of the inferred metabolic pathways, we used EC2KEGG (available at http://sourceforge.net/projects/ec2kegg) to compute the false discovery rate (FDR) of each pathway [31]. Any pathway with an FDR adjusted *p*-value below 0.05 is considered statistically significant. Currently, there is only one reference genome for the genus *Herbaspirillum* in KEGG: *H. seropedicae*. Hence, the genome of *H. seropedicae* was chosen as the reference genome for statistical evaluation.

## Results and discussion

### Function annotations and pathway analysis

Our combined analyses using EFICAz, MetaCyc Blast and KEGG Blast identified 2293 genes in RV1423 that potentially encode 986 different enzymatic activities as specified by their EC numbers with all four classes ascertained [see Additional file 1]. These enzymes were mapped to 134 unique metabolic pathways defined in KEGG. In the EC2KEGG analysis, we found 110 of these metabolic pathways that were significantly over-represented when compared to the reference genome [see Additional file 2]. Naphthalene degradation was among the over-represented pathways, so in this analysis we further examined the enzymes identified in this pathway. As shown on Table 1, our results suggested that RV1423 may encode 8 enzymes involved in the naphthalene degradation pathway, whereas only one of these enzymes is likely encoded by the reference genome (EC number 1.1.1.1). In RV1423, RAST annotation yielded four genes that were identified to encode gentisate 1,2-dioxygenase (1.13.11.4; gentisate 12C2-dioxygenase), which is an enzyme involved in the gentisate pathway for salicylate metabolism. Naphthalene can be degraded to form salicylate, which can then be further metabolized by the gentisate pathway [32]. Our finding of such gene duplication is consistent with a previous study suggesting the importance of the gentisate pathway for naphthalene degradation [27]. This suggests a possible mechanism by which naphthalene is degraded in RV1423 that is absent in the reference genome, *H. seropedicae*.

**Table 1:** List of predicted enzymes in the naphthalene degradation pathway

EC Number	Enzyme Description	Number of Genes	
		RV1423	H. seropedicae
1.1.1.1	Alcohol dehydrogenase	3	3
1.2.1.65	Salicylaldehyde dehydrogenase	2	0
1.3.1.29	Cis-1,2-dihydro-1,2-dihydroxynaphthalene dehydrogenase	1	0
1.13.11.56	1,2-dihydroxynaphthalene dioxygenase	1	0
1.14.13.172	Salicylate 1-monooxygenase	5	0
1.14.12.12	Naphthalene 1,2-dioxygenase	3	0
4.1.2.45	Trans-o-hydroxybenzylidenepyruvate hydratase-aldolase	1	0
5.99.1.4	2-hydroxychromene-2-carboylate isomerase	2	0

Genes with putative function (EC number and enzyme description) were identified in RV1423 along with the number of genes encoding these enzymes in the RV1423 and *H. seropedicae* genomes

### Runtime analysis

The whole genome analysis of RV1423 containing 5649 proteins took approximately 8 h when selecting all of the currently available PSAT tools while running on 60 cores from 5 compute nodes on the PSAT cluster. In order to assess the throughput of PSAT, we performed a runtime analysis of EFICAz and KEGG Blast for four sample genomes in addition to RV1423. This was performed in parallel and serial modes using 60 cores from 5 compute nodes and a single core on a single node on the PSAT cluster, respectively. Table 2 shows that PSAT running in parallel mode on a multi-core Linux cluster scales well with increasingly large datasets, and is at least 30 times faster than running in serial mode for a set of 100 randomly selected proteins. Thus, enzyme prediction provided by the PSAT server is high throughput, enabling rapid enzyme predictions over whole-genome peptide fasta sequence sets.

**Table 2:** Runtime analyses of back-end processing

Genome	Number of Proteins	Parallel (HH:MM:SS)	Serial (HH:MM:SS)
*Alcanivorax borkumensis* SK2	2755	1:57:16	2:27:00
*Marinobacter aquaeolei*	3858	2:35:41	2:31:10
*Gordonia Sp*. KTR9	4741	3:33:57	2:24:24
*Pseudomonas mendocina*	4958	3:35:34	2:30:09

Jobs were run in parallel and serial modes using four different genomes and 100 proteins randomly selected from their corresponding genome, respectively

## Conclusion

The PSAT server is a centralized online service offering several function-based protein annotation resources, and is capable of performing high-throughput protein sequence annotation and analysis. PSAT provides a convenient way for scientists to derive enzyme functions of proteins and to identify their corresponding metabolic pathways defined in the KEGG database. The above-described case study identified a list of putative metabolic pathways in *Herbaspirillum sp*. strain RV1423, thus demonstrating that PSAT is capable of deriving biological information that is consistent with the existing literature. Hence, the predicted protein functions, including the enzymatic activities and catabolic potentials of the query proteins from RV1423, may warrant further experimental studies. Because PSAT can rapidly provide de novo sequence-based enzyme prediction over whole proteomes by combining multiple methods, it can assist in the identification of genes, reactions, or pathways that may help explain an observed phenotype.

PSAT aims to improve the scalability and usability of each individual sequence annotation tool by adding a distributed computing component to the architecture to run multiple processes in parallel. As demonstrated in this case study, PSAT was able to process a large set of proteins predicted from a whole genome in an efficient and systematic manner. Furthermore, additional annotation sources can easily be adapted and executed in parallel under the PSAT architecture. Future development of the PSAT platform will primarily depend on the demand of its users and its role in empowering subsequent scientific discoveries.

## Availability and requirements

- **Project name:** Protein Sequence Annotation Tool (PSAT)
- **Project home page:**http://psat.llnl.gov/psat
- **Operating system(s):** Platform independent
- **Programming language:** Python
- **Other requirements:** Chrome (41.0.2218.0) or Firefox (33.1)
- **License:** None required
- **Any restrictions to use by non-academics:** None

## Additional files

Additional file 1:
**Summary data for PSAT analysis of RV1423.** (XLSX 1543 kb) Additional file 2:
**The EC2KEGG output for the RV1423 analysis sorted in ascending order by the FDR value.** (XLSX 58 kb)

## Abbreviations

PSAT: protein sequence annotation tool
HTTP: hypertext transfer protocol
IGS: Institute for Genome Sciences
NCBI: National Center for Biotechnology Information
EC: enzyme commission
